# The effects of β-hydroxy-β-methylbutyrate or HMB-rich nutritional supplements on sarcopenia patients: a systematic review and meta-analysis

**DOI:** 10.3389/fmed.2024.1348212

**Published:** 2024-07-12

**Authors:** Hai Su, Haojing Zhou, Yichen Gong, Sicheng Xiang, Weijie Shao, Xinzheng Zhao, Hao Ling, Guoqian Chen, Peijian Tong, Ju Li

**Affiliations:** The First Affiliated Hospital of Zhejiang Chinese Medical University (Zhejiang Provincial Hospital of Chinese Medicine), Hangzhou, China

**Keywords:** β-hydroxy-β-methylbutyrate, HMB, sarcopenia, meta-analysis, muscle

## Abstract

**Background:**

Sarcopenia is a progressive, systemic skeletal muscle disorder. Resistance exercise and physical activity have been proven effective in its treatment, but consensus on pharmacological interventions has not yet been reached in clinical practice. β-Hydroxy-β-methylbutyrate (HMB) is a nutritional supplement that has demonstrated favorable effects on muscle protein turnover, potentially contributing to beneficial impacts on sarcopenia.

**Aim:**

To assess the potential positive effects of HMB or HMB-containing supplements on individuals with sarcopenia, a systematic review and meta-analysis was conducted.

**Methods:**

A systematic review and meta-analysis were conducted on randomized controlled trials (RCTs) examining the treatment of sarcopenia with HMB. Two assessors independently conducted screening, data extraction, and bias risk assessment. Outcome data were synthesized through a random-effects model in meta-analysis, using the mean difference (MD) as the effect measure.

**Results:**

A meta-analysis was conducted on six studies. HMB or HMB-rich nutritional supplements showed a statistically significant difference in Hand Grip Strength (HGS) for sarcopenia patients [MD = 1.26, 95%CI (0.41, 2.21), *p* = 0.004], while there was no statistically significant difference in Gait Speed (GS) [MD = 0.04, 95%CI (−0.01, 0.08), *p* = 0.09], Fat Mass (FM) [MD = −0.18, 95%CI (−0.38, 0.01), *p* = 0.07], Fat-Free Mass (FFM) [MD = 0.09, 95%CI (−0.23, 0.42), *p* = 0.58], and Skeletal Muscle Index (SMI) [MD = 0.01, 95%CI (−0.00, 0.01), *p* = 0.13].

**Conclusion:**

HMB or HMB-rich nutritional supplements are beneficial for muscle strength in sarcopenia patients. However, there is limited evidence demonstrating significant effects on both muscle strength and physical performance in sarcopenia individuals. HMB may be considered as a treatment option for sarcopenia patients.

**Systematic review registration:**

CRD42024512119.

## 1 Introduction

Sarcopenia is clinically defined as a progressive, systemic musculoskeletal disorder (1). It is widely acknowledged that sarcopenia is more prevalent in the elderly population. In fact, the decline in muscle mass typically initiates around the age of 40 (2). Consequently, the adverse impacts of sarcopenia on quality of life, healthcare demands, incidence, and mortality may affect middle-aged and older individuals (3). Research suggests that this condition is commonly observed in both males and females, with an estimated prevalence of around 10% in individuals aged 65 and above (4). Another study indicates that sarcopenia affects 10–16% of the elderly population globally (5). With its increasing prevalence and the global trend of population aging, sarcopenia has become a focal point in contemporary clinical epidemiology (6). The diagnosis of sarcopenia comprises three aspects: muscle strength, muscle mass, and physical performance. Muscle strength is assessed through hand grip strength (HGS), typically measured using a dynamometer. Muscle mass encompasses measures such as fat mass (FM), fat-free mass (FFM), and skeletal muscle mass index (SMI), which can be assessed using dual-energy X-ray absorptiometry (DEXA), bioelectrical impedance analysis (BIA), ultrasound, magnetic resonance imaging (MRI), and computed tomography (CT), among others. Physical performance is often evaluated through gait speed (GS), which can be measured using an automated timer (6, 7). Currently, the preferred treatment for sarcopenia in clinical practice remains resistance exercise therapy (8), as there is still no evidence supporting pharmaceutical interventions for treating sarcopenia (9). While resistance exercise has demonstrated significant efficacy for sarcopenia patients, nutritional intervention is often the primary treatment for those unable to engage in physical activity (10).

β-Hydroxy-β-Methylbutyrate (HMB) is a nutritional supplement, a bioactive metabolite formed from the breakdown of the essential branched-chain amino acid leucine (11). HMB plays various roles in the human body, with its most crucial functions involving protein metabolism, insulin activity, and skeletal muscle hypertrophy (12). HMB can stimulate the mechanistic Target of Rapamycin (mTOR) signaling pathway, promoting protein synthesis, while inhibiting protein degradation through the attenuation of the proteasome pathway (12, 13). It also enhances muscle membrane integrity, making it a recognized key regulator of muscle protein synthesis metabolism in clinical settings (14, 15). Studies suggest that daily supplementation of HMB can increase muscle mass and reduce muscle damage in the elderly (16–18). Even for bedridden healthy elderly individuals without resistance training, HMB supplementation has shown positive effects in maintaining muscle mass (19), indicating its potential as an effective nutritional intervention for patients unable to engage in physical activity, such as those in critical conditions (20–22).

However, there is currently no high-quality clinical evidence supporting whether HMB has a beneficial therapeutic effect on sarcopenia patients. Assuming that HMB is beneficial for patients with sarcopenia. Therefore, we conducted a systematic review and meta-analysis to investigate whether the use of HMB alone or in protein supplements containing HMB has favorable impacts on sarcopenia patients.

## 2 Methods

### 2.1 Search strategy

This study strictly adheres to the guidelines outlined in the Preferred Reporting Items for Systematic Review and Meta-Analysis Protocols (PRISMA-P) (23). Two researchers systematically conducted searches across four electronic databases—Medline/PubMed, Cochrane Library, Embase, and Web of Science—employing a comprehensive strategy that integrated both free terms and database-specific subject terms. This approach aimed to thoroughly retrieve relevant papers pertinent to this study. In cases of disagreement, a third researcher was responsible for facilitating discussions and reaching a consensus. The last search was conducted on October 24, 2023. The Medline/PubMed search example is as follows: “sarcopeni*”[Title/Abstract] OR “presarcopeni*”[Title/Abstract] OR “pre sarcopeni*”[Title/Abstract] OR “Sarcopenia”[MeSH Terms]) AND (“HMB”[Title/Abstract] OR “beta-hydroxy-beta-methylbutyrate”[Title/Abstract] OR “β-hydroxy-β-methylbutyrate”[Title/Abstract].

### 2.2 Study selection criteria

All retrieved papers are stored in EndNote X9. Two researchers individually conducted screening based on titles and abstracts to identify relevant literature for the study. In instances of disagreement between the two researchers, a third researcher facilitated discussions to reach a consensus. Inclusion criteria for the study were as follows: (1) Diagnosis of sarcopenia according to the European Working Group on Sarcopenia in Older People (EWGSOP), Asian Working Group on Sarcopenia (AWGS) (6, 24), consensus, or any other definition used by the authors of the original study; (2) Randomized controlled trial (RCT); (3) Use of HMB or HMB-rich preparations in the intervention group. Exclusion criteria were as follows: (1) Animal or *in vitro* studies; (2) Studies where data cannot be accurately extracted or where data is missing.

### 2.3 Date collection, and data extraction

Two researchers independently extracted data from the included study by reading the full text and entered the extracted data into a pre-designed data extraction form. In cases of discrepancies during the extraction process, a third researcher facilitated discussions to reach a consensus. The extracted study features included the authors, publication year, publication date, study location, research design, diagnostic criteria, basic characteristics and numbers of study subjects, interventions for the intervention and control groups, follow-up duration, and outcome data.

### 2.4 Quality assessment

Two researchers independently assessed the quality of the studies using the standards outlined in the Cochrane Handbook for Systematic Reviews of Interventions (25). These standards encompassed the following seven criteria: random sequence generation, allocation concealment, blinding of participants and personnel, blinding of outcome data, addressing incomplete outcome data, selective reporting, and other biases.

### 2.5 Statistical analysis

This study utilized the statistical software RevMan 5.3 for conducting meta-analyses. As all included outcome measures were continuous variables, the mean difference (MD) was chosen as the effect size, with each effect size accompanied by a point estimate and a 95% confidence interval (CI). For studies that provided data in terms of median and range, the data were transformed according to the appropriate formulas before being included in the pooled analysis (26, 27). Heterogeneity was assessed through a *χ*^2^ test (α = 0.1) and complemented by the I2 statistic. If the I2 value was less than 50%, a fixed-effects model was employed for the meta-analysis. If the I2 value was 50% or greater, indicating substantial statistical heterogeneity among the study results, further analysis of the sources of heterogeneity was conducted. After excluding apparent clinical and methodological heterogeneity, a random-effects model was then applied for the meta-analysis. Sensitivity analysis entails the stepwise exclusion of individual study results, followed by a reiteration of the meta-analysis, and an evaluation of the disparities between the revised outcomes and the initial combined results.

## 3 Results

### 3.1 Search result

**Figure 1:**
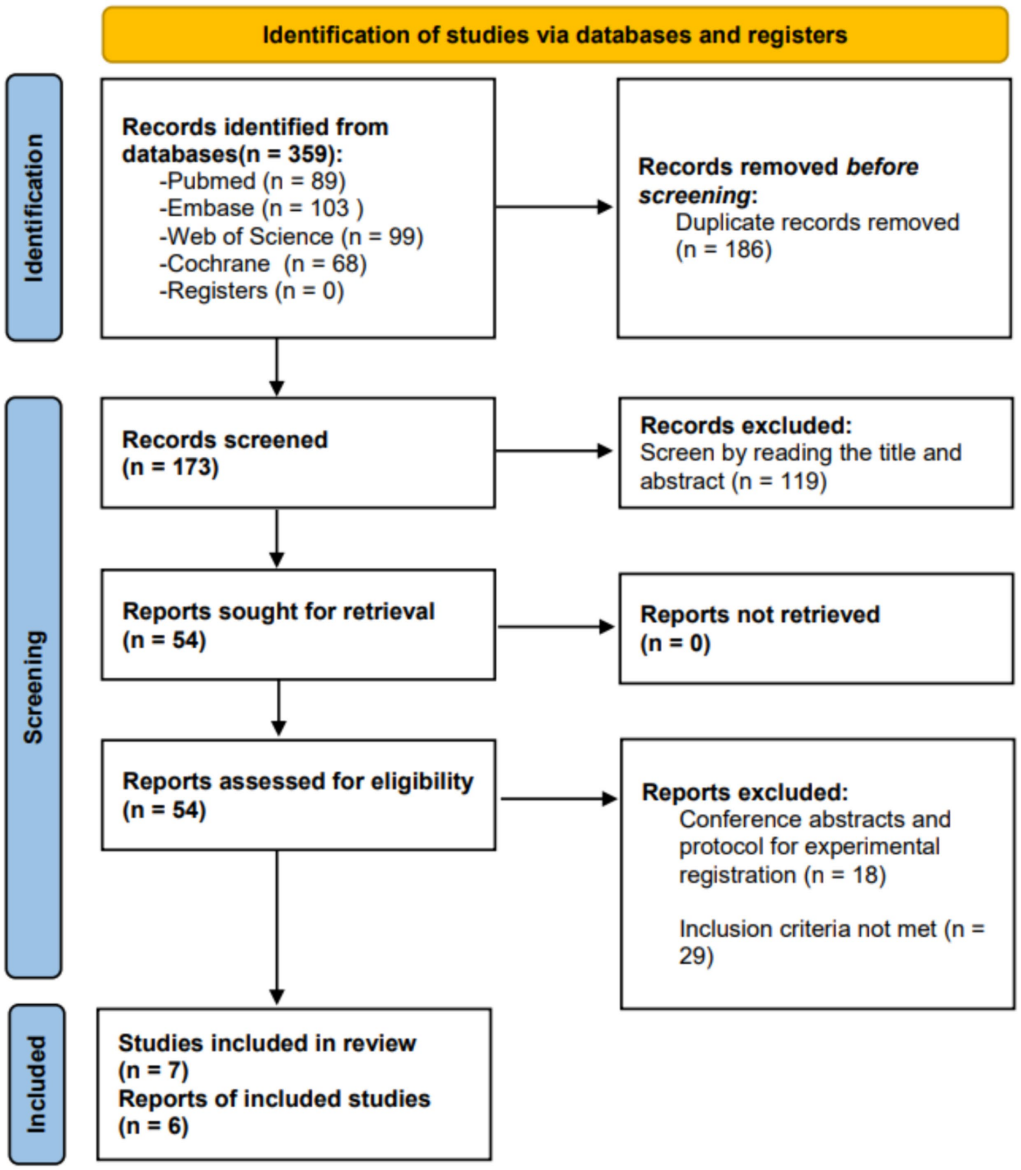
PRISMA screening procedure.

We retrieved a total of 359 studies. After removing duplicate studies, we identified 173 studies for title and abstract screening. Through the screening of title and abstract, we confirmed 54 studies and conducted a full-text review. Among them, 30 studies did not meet the inclusion criteria, and 18 studies were classified as conference abstracts or experimental registration plans. Finally, we included 7 studies in the systematic review. However, one study (28) had limited outcome indicators, allowing for quality assessment but preventing inclusion in the meta-analysis. Therefore, only 6 studies were included in the meta-analysis. Figure 1 illustrates the study selection process and search results.

### 3.2 Study characteristics

Seven studies met the inclusion criteria for quality assessment (28–34). All seven studies utilized established diagnostic criteria and included patients diagnosed with sarcopenia, such as the criteria from the EWGSOP and the AWGS. In one study (29), participants were post-liver transplant patients with concomitant sarcopenia, while another study (33) included sarcopenia patients with hip fractures. The HMB intake was 1.2 g/d in two studies (28, 34), and 3 g/d in the remaining five studies (29–33). The intervention duration in one study was only during the hospitalization period (33), while the nutrition intervention duration in the other six studies was 12 weeks. Detailed characteristics of the studies are presented in Table 1.

**Table 1:** Characteristics of the included studies.

Study	Region	Subject characteristics	Sex	Sarcopenia definition	β-Hydroxy-β-methylbutyrate dose (per day)	Duration	Primary outcome	Secondary outcome
Lattanzi et al. (29)	Italy	Had a liver transplant	Male	ASMI or FFMI <5th percentile	3 g/d	12 weeks	Muscle mass	Muscle function, safety profile
Cramer et al. (31)	United States	Men and women 65 years and older from 8 countries across Europe and North America With both malnutrition and sarcopenia were enrolled	Male and female	EWGSOP	3 g/d	12 weeks/24 weeks	Isokinetic peak torque (PT, Nm) leg strength	Weight, LMM, TLMM, grip strength, gait speed, and product compliance
Nasimi et al. (32)	Iran	Aged 65 years and older and had the sarcopenia criteria	Male and female	AWGS	3 g/d	12 weeks	Lean mass and appendicular lean mass	HGS, gait speed, anabolic, metabolic, Inflammatory, oxidative stress biomarkers
Malafarina et al. (33)	Spain	Combined with a hip fracture	Male and female	EWGSOP	3 g/d	Duration of hospital stay	MNA-SF	Haemoglobin, Total protein, Albumin, Prealbumin, Total, Cholesterol, Triglycerides, IL-1, IL-6, Insulin, HOMA, Gait-speed, Hand-grip, muscle mass, appendicular lean mass, Skeletal Muscle Mass, fatty mass, Fat free mass, appendicular skeletal muscle mass
Osuka et al. (34)	Japan	This survey targeted older women, aged 65–79 y, living in the Itabashi Ward, located in the northwest area of 23 special wards in Tokyo, Japan	Female	AWGS	1.2 g/d	12 weeks	Muscle mass	Muscle strength, physical performance, functional capacity, blood markers, habitual dietary intake, and habitual physical activity levels
Yang et al. (30)	China	Age ≥ 60 years, diagnosed with sarcopenia according to the Asian Working Group for Sarcopenia	Male and female	AWGS	3 g/d	12 weeks	Handgrip strength	Gait speed, five-time chair stand test, body compositions, and anthropometric measurements
Osuka et al. (28)	Japan	Age ≥ 65 years, with sarcopenia according to the Asian Working Group for Sarcopenia	Female	AWGS	1.2 g/d	12 weeks	Muscle quality	Subcutaneous fat, Rectus femoris, Vastus intermedius, Quadriceps femoris

### 3.3 Quality assessment and publication bias

**Figure 2:**
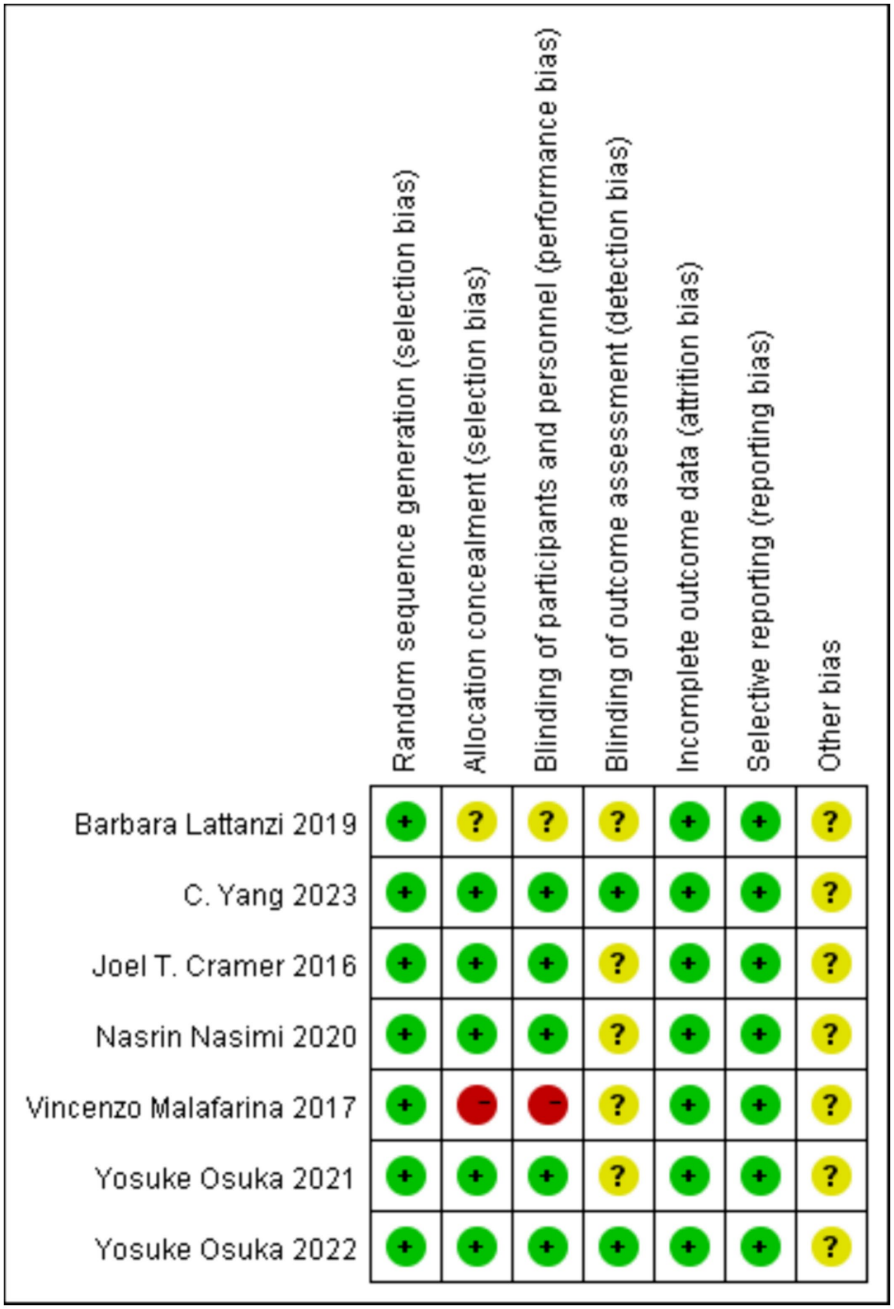
Risk of bias of included studies.

No study was considered at low risk across all items. All participants met the criteria for random allocation, but it remains unclear whether all trials adhered to all quality assessment criteria (Figure 2). As the literature included in this study is less than 10 articles, publication bias detection was not performed.

### 3.4 Study results

Regardless of the measurement approach used, our primary outcome measure is the patient’s HGS, and secondary outcome measures include GS, FM, FFM, and SMI. One study (34) included four treatment groups: exercise plus HMB, exercise plus placebo, education plus HMB, and education plus placebo. Therefore, the data from this study were divided into two groups for analysis. Another study (31) categorized sarcopenia patients into severe and mild–moderate groups, both undergoing intervention with HMB nutritional supplements. Consequently, the data from this study were also divided into two groups for analysis. The intervention period in one study extended from admission to discharge, in contrast to the remaining studies which uniformly had a 12-week intervention duration. Additionally, one study implemented a combined approach for interventions, while the others exclusively administered either HMB or HMB-rich nutritional supplements. In one study, the dosage of HMB was 1.2 g, while in the remaining studies, the dosage of HMB was 3 g. Three studies involved populations from Asia, and three studies involved populations from Europe. Two studies the patient combined with other diseases, and four studies the patient without other diseases. These variations may introduce heterogeneity into the analysis. Therefore, we conducted subgroup analyses based on intervention duration, intervention method, population, the dosage of HMB, and combined with other diseases for HGS, GS, FM, and FFM data during the analysis, aiming to reduce the heterogeneity in the article. The results of sensitivity analyses can be found in Supplementary materials.

#### 3.4.1 HGS

**Figure 3:**
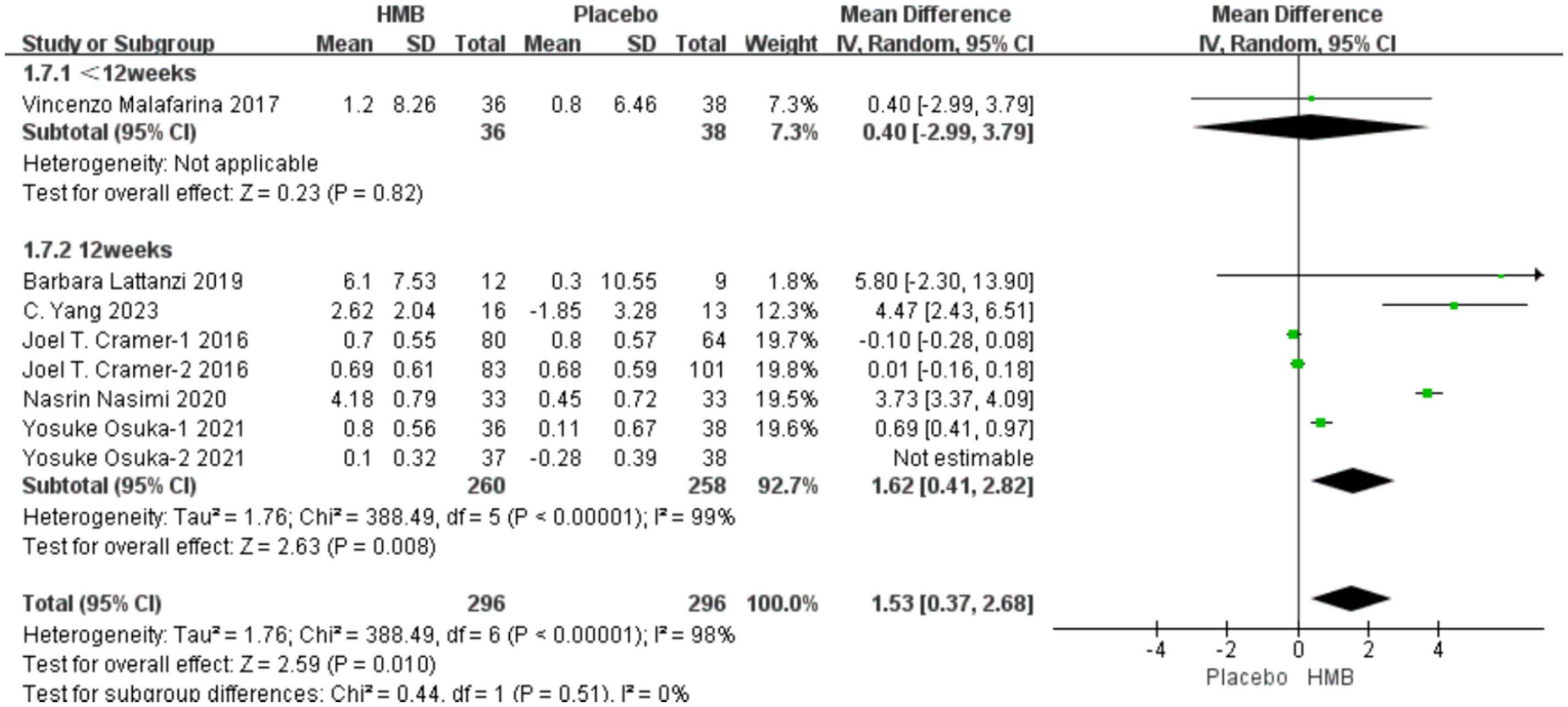
Forest plot for changes in hand grip strength. The horizontal lines represent 95% CI. The diamond data markers indicate the mean difference (MD) of HMB supplementation on hand grip strength.

A total of 6 studies, involving 667 patients, were included in the analysis. According to the random-effects model meta-analysis results, when the intervention duration was less than 12 weeks, there was no statistically significant difference in HGS between the HMB group and the control group [MD = 0.40, 95%CI (−2.99, 3.79), *p* = 0.82] (Figure 3). However, with an intervention duration of 12 weeks, a statistically significant difference in HGS emerged between the HMB group and the control group [MD = 1.31, 95%CI (0.43, 2.18), *p* = 0.003, I2 = 98%] (Figure 3). When the intervention involved the combination of HMB with other methods, there was a statistically significant difference in HGS between the HMB group and the control group [MD = 0.51, 95% CI (0.21, 0.81), *p* = 0.0008, I2 = 72%]. Similarly, when the intervention consisted solely of HMB, a statistically significant difference in HGS between the HMB group and the control group was also observed [MD = 1.78, 95% CI (0.31, 3.25), *p* = 0.02, I2 = 99%]. There was a significant difference in HGS between the HMB group and the control group when the population was from Asia [MD = 0.07, 95% CI (0.02, 0.13), *p* = 0.01, I2 = 99%]. However, when the population was from Europe, no statistically significant difference in HGS was observed between the HMB group and the control group [MD = −0.40, 95% CI (−0.17, 0.09), *p* = 0.05, I2 = 0%]. There was a significant difference in HGS between the HMB group and the control group when the dosage of HMB was less than 3 g [MD = 0.51, 95% CI (0.21, 0.81), *p* = 0.0008, I2 = 72%]. Similarly, a significant difference in HGS was observed between the HMB group and the control group when the dosage of HMB was 3 g [MD = 2.07, 95% CI (0.53, 3.60), *p* = 0.008, I2 = 99%]. There was a significant difference in HGS between the HMB group and the control group when the patient without other diseases [MD = 1.26, 95% CI (0.38, 2.13), *p* = 0.005, I2 = 99%]. However, when the patient combined with other diseases, no statistically significant difference in HGS was observed between the HMB group and the control group [MD = 1.79, 95% CI (−2.84, 6.43), *p* = 0.45, I2 = 31%] (Details can be found in Supplementary materials). Overall, the results indicated a statistically significant difference in HGS between the HMB group and the control group [MD = 1.26, 95%CI (0.41, 2.21), *p* = 0.004, I2 = 98%] (Figure 3).

#### 3.4.2 GS

**Figure 4:**
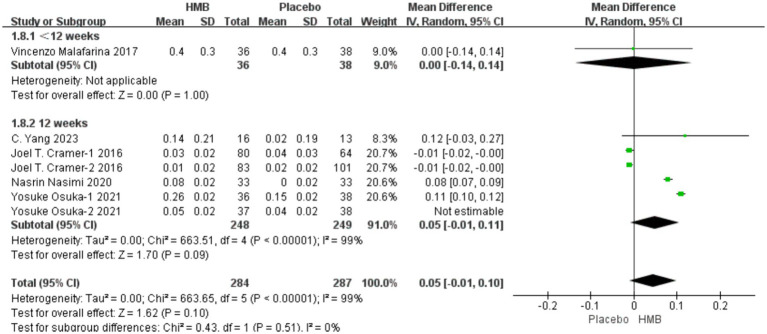
Forest plot for changes in gait speed. The horizontal lines represent 95% CI. The diamond data markers indicate the mean difference (MD) of HMB supplementation on gait speed.

A total of 5 studies, including 646 patients, were included in the analysis. According to the random-effects model meta-analysis results, when the intervention duration was less than 12 weeks, there was no statistically significant difference in GS between the HMB group and the control group [MD = 0.00, 95%CI (−0.14, 0.14), *p* = 1.00] (Figure 4). With an intervention duration of 12 weeks, there was no statistically significant difference in GS between the HMB group and the control group [MD = 0.04, 95%CI (−0.00, 0.09), *p* = 0.08, I2 = 99%] (Figure 4). When the intervention involved the combination of HMB with other methods, there was no statistically significant difference in GS between the HMB group and the control group [MD = 0.06, 95% CI (−0.04, 0.16), *p* = 0.23, I2 = 100%]. Similarly, when the intervention consisted solely of HMB, there was no statistically significant difference in GS between the HMB group and the control group [MD = 0.03, 95% CI (−0.02, 0.07), *p* = 0.29, I2 = 99%]. There was a significant difference in GS between the HMB group and the control group when the population was from Asia [MD = 0.07, 95% CI (0.02, 0.13), *p* = 0.01, I2 = 99%]. However, when the population was from Europe, no statistically significant difference in GS between the HMB group and the control group was observed [MD = −0.40, 95%CI (−0.17, 0.09), *p* = 0.52, I2 = 0%]. However, the sensitivity analyses did not support this conclusion, and it is possible that there were false positives. There was no significant difference in GS between the HMB group and the control group when the dosage of HMB was less than 3 g [MD = 0.06, 95% CI (−0.04, 0.16), *p* = 0.23, I2 = 100%]. When the dosage of HMB was 3 g, there was no significant difference in GS between the HMB group and the control group [MD = 0.03, 95% CI (−0.02, 0.07), *p* = 0.29, I2 = 99%]. There was no significant difference in GS between the HMB group and the control group when the patient without other diseases [MD = 0.04, 95% CI (0.00, 0.09), *p* = 0.08, I2 = 99%]. When the patient combined with other diseases, no statistically significant difference in GS was observed between the HMB group and the control group [MD = 0.00, 95% CI (−0.14, 0.14), *p* = 1.00] (Details can be found in Supplementary materials). Overall, the results indicated a statistically non-significant difference in GS between the HMB group and the control group [MD = 0.04, 95%CI (−0.01, 0.08), *p* = 0.09, I2 = 99%] (Figure 4).

#### 3.4.3 FM

**Figure 5:**
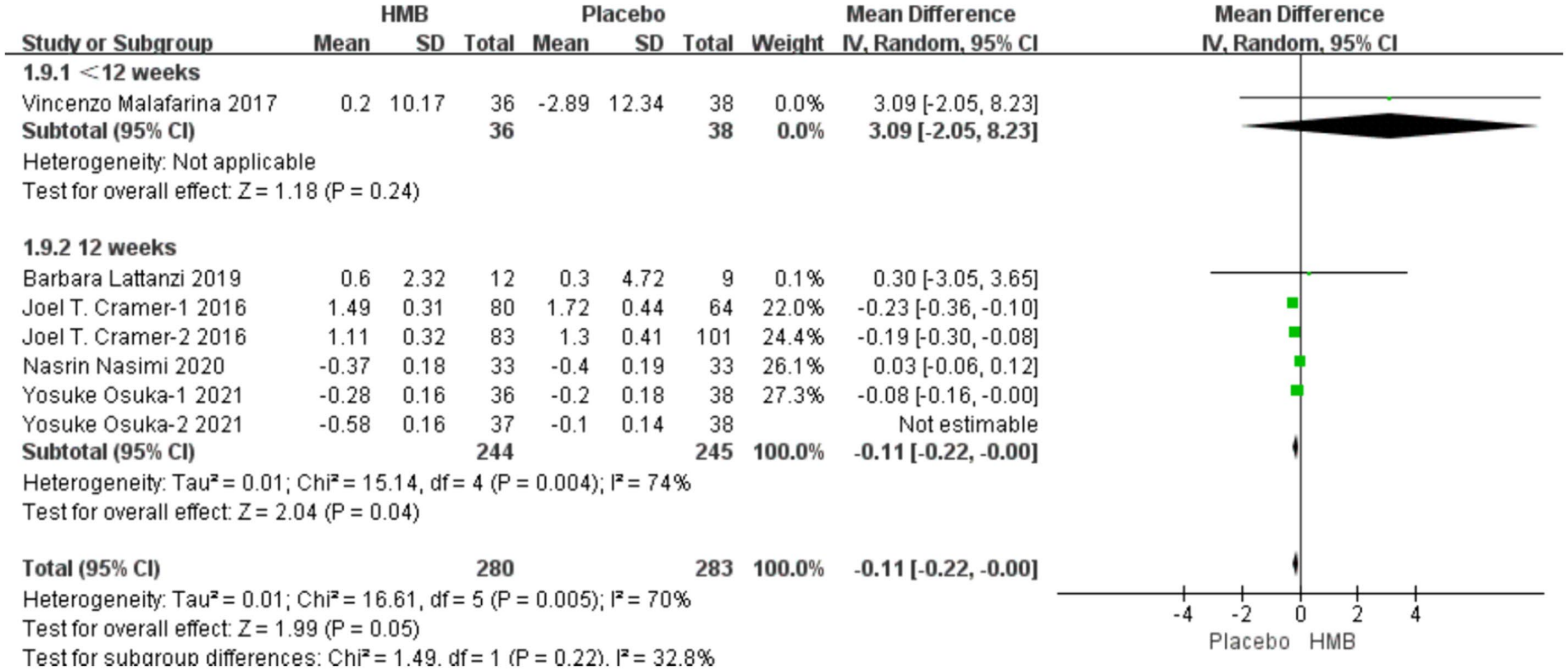
Forest plot for changes in fat mass. The horizontal lines represent 95% CI. The diamond data markers indicate the mean difference (MD) of HMB supplementation on fat mass.

A total of 5 studies, including 638 patients, were included in the analysis. According to the random-effects model meta-analysis results, when the intervention duration was less than 12 weeks, there was no statistically significant difference in FM between the HMB group and the control group [MD = 3.09, 95%CI (−2.05, 8.23), *p* = 0.24] (Figure 5). With an intervention duration of 12 weeks, there was no statistically significant difference in FM between the HMB group and the control group [MD = −0.19, 95%CI (−0.39, 0.01), *p* = 0.06, I2 = 95%] (Figure 5). When the intervention involved the combination of HMB with other methods, there was no statistically significant difference in FM between the HMB group and the control group [MD = −0.28, 95%CI (−0.67, 0.11), *p* = 0.16, I2 = 98%]. Similarly, when the intervention consisted solely of HMB, there was no statistically significant difference in FM between the HMB group and the control group [MD = −0.12, 95%CI (−0.29, 0.04), *p* = 0.15, I2 = 76%]. For populations from Asia, there was a significant difference in FM between the HMB group and the control group [MD = 0.07, 95% CI (0.01, 0.13), *p* = 0.03, I2 = 99%]. However, for populations from Europe, there was no statistically significant difference in FM between the HMB group and the control group [MD = −0.40, 95%CI (−0.17, 0.09), *p* = 0.54, I2 = 0%]. There was no significant difference in FM between the HMB group and the control group when the dosage of HMB was less than 3 g [MD = −0.28, 95% CI (−0.67, 0.11), *p* = 0.16, I2 = 98%]. Similarly, when the dosage of HMB was 3 g, there was no significant difference in FM between the HMB group and the control group [MD = −0.12, 95% CI (−0.29, 0.04), *p* = 0.07, I2 = 76%]. There was no significant difference in FM between the HMB group and the control group when the patient without other diseases [MD = −0.19, 95% CI (−0.39, 0.1), *p* = 0.06]. When the patient combined with other diseases, no statistically significant difference in FM was observed between the HMB group and the control group [MD = 1.13, 95% CI (−1.68, 3.94), *p* = 0.37, I2 = 0%] (Details can be found in Supplementary materials). Overall, the results indicated no statistically significant difference in FM between the HMB group and the control group [MD = −0.18, 95%CI (−0.38, 0.01), *p* = 0.07, I2 = 94%] (Figure 5).

#### 3.4.4 FFM

**Figure 6:**
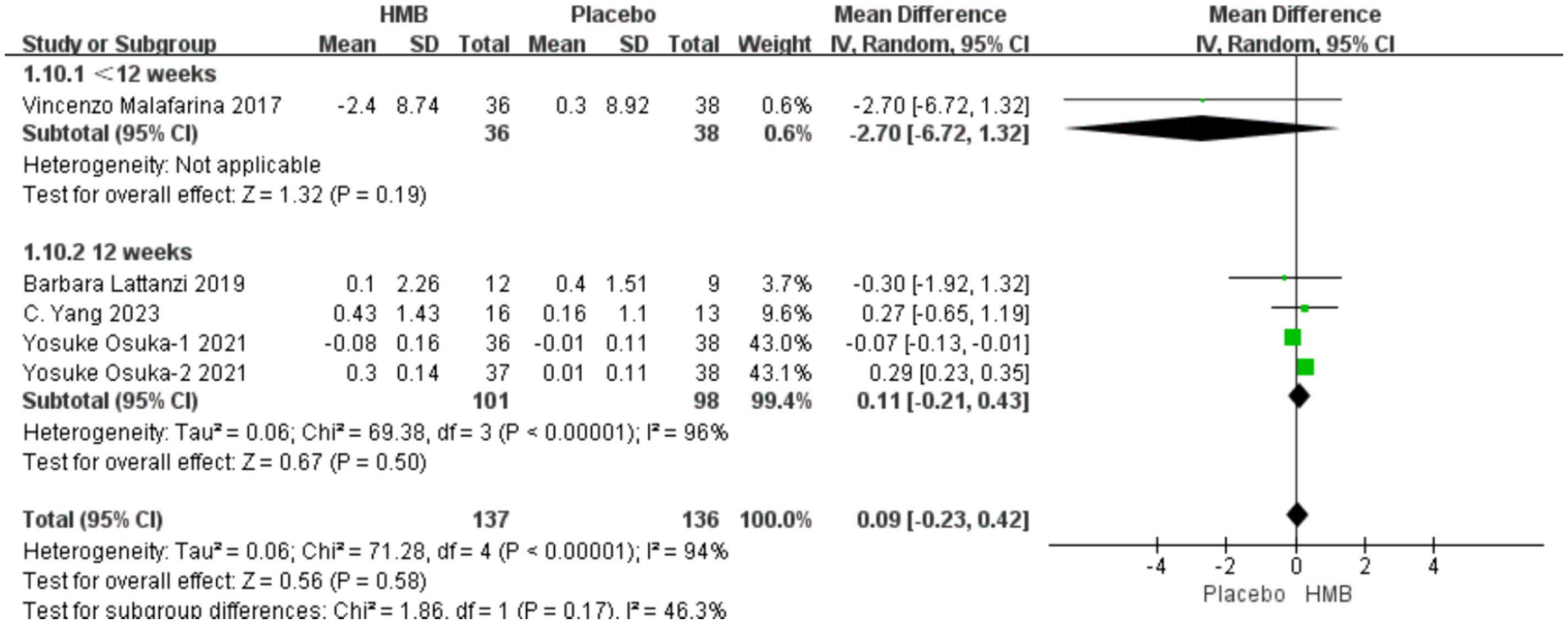
Forest plot for changes in fat free mass. The horizontal lines represent 95% CI. The diamond data markers indicate the mean difference (MD) of HMB supplementation on fat free mass.

A total of 4 studies, including 273 patients, were included in the analysis. According to the random-effects model meta-analysis results, when the intervention duration was less than 12 weeks, there was no statistically significant difference in FFM between the HMB group and the control group [MD = −2.70, 95%CI (−6.72, 1.32), *p* = 0.19] (Figure 6). With an intervention duration of 12 weeks, there was no statistically significant difference in FFM between the HMB group and the control group [MD = 0.11, 95%CI (−0.21, 0.43), *p* = 0.50, I2 = 96%] (Figure 6). When the intervention involved the combination of HMB with other methods, there was no statistically significant difference in FFM between the HMB group and the control group [MD = 0.11, 95%CI (−0.24, 0.46), *p* = 0.54, I2 = 99%]. Similarly, when the intervention consisted solely of HMB, there was no statistically significant difference in FFM between the HMB group and the control group [MD = 0.20, 95%CI (−0.59, 0.99), *p* = 0.62, I2 = 0%]. There was no significant difference in FFM between the HMB group and the control group in the Asian population [MD = 0.13, 95% CI (−0.20, 0.46), *p* = 0.45, I2 = 97%]. Similarly, in the European population, there was no statistically significant difference in FFM between the HMB group and the control group [MD = −0.76, 95%CI (−2.62, 1.09), *p* = 0.42, I2 = 15%]. There was no significant difference in FFM between the HMB group and the control group when the dosage of HMB was less than 3 g [MD = 0.11, 95% CI (−0.24, 0.46), *p* = 0.54, I2 = 99%]. Similarly, when the dosage of HMB was 3 g, there was no significant difference in FFM between the HMB group and the control group [MD = 0.20, 95% CI (−0.59, 0.99), *p* = 0.62, I2 = 0%] There was no significant difference in FFM between the HMB group and the control group when the patient without other diseases [MD = 0.13, 95% CI (−0.20, 0.46), *p* = 0.58, I2 = 94%]. When the patient combined with other diseases, no statistically significant difference in FFM was observed between the HMB group and the control group [MD = −0.76, 95% CI (−2.62, 1.09), *p* = 0.37, I2 = 15%] (Details can be found in Supplementary materials). Overall, the results indicated no statistically significant difference in FFM between the HMB group and the control group [MD = 0.09, 95%CI (−0.23, 0.42), p = 0.58, I2 = 94%] (Figure 6).

#### 3.4.5 SMI

**Figure 7:**
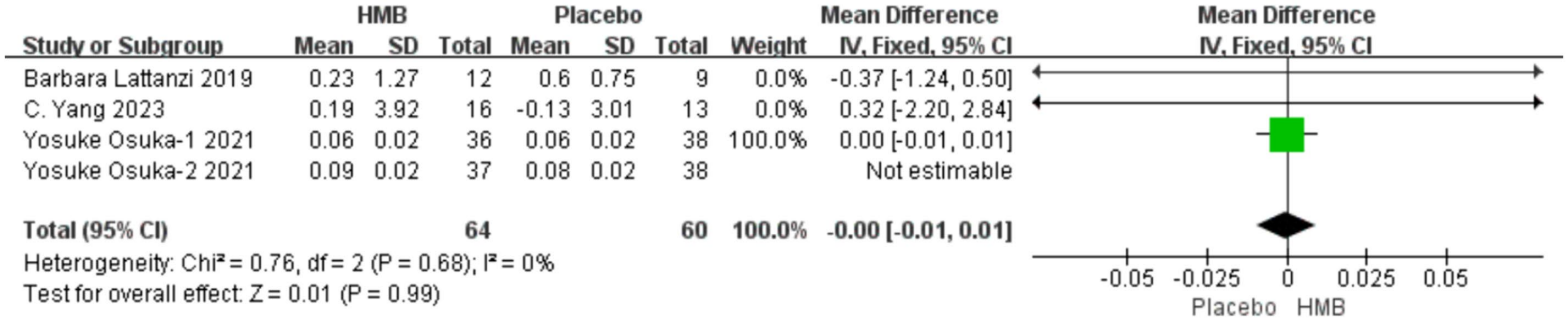
Forest plot for changes in skeletal muscle index. The horizontal lines represent 95% CI. The diamond data markers indicate the mean difference (MD) of HMB supplementation on skeletal muscle index.

A total of 3 studies, including 199 patients, were included in the analysis. According to the random-effects model meta-analysis results, there was no statistically significant difference in SMI between the HMB group and the control group [MD = 0.01, 95%CI (−0.00, 0.01), *p* = 0.13] (Figure 7). When the intervention involved the combination of HMB with other methods, there was no statistically significant difference in SMI between the HMB group and the control group [MD = 0.01, 95%CI (−0.00, 0.01), *p* = 0.54, I2 = 57%]. Similarly, when the intervention consisted solely of HMB, there was no statistically significant difference in SMI between the HMB group and the control group [MD = 0.01, 95%CI (−0.00, 0.01), *p* = 0.13, I2 = 3%]. There was no significant difference in SMI between the HMB group and the control group in populations from Asia [MD = 0.01, 95% CI (−0.00, 0.01), *p* = 0.12, I2 = 16%]. Similarly, in populations from Europe, there was no statistically significant difference in SMI between the HMB group and the control group [MD = −0.37, 95%CI (−1.24, 0.50), p = 0.13, I2 = 3%]. When the dosage of HMB was less than 3 g, there was no significant difference in SMI between the HMB group and the control group [MD = 0.01; 95% CI (−0.00, 0.01); p = 0.54; I2 = 57%]. When the dosage of HMB was 3 g, there was no significant difference in SMI between the HMB group and the control group [mean difference (MD) = 0.01; 95% confidence interval (CI) = (−0.00, 0.01); p = 0.13; I2 = 3%]. There was no significant difference in SMI between the HMB group and the control group when the patient without other diseases [MD = 0.01, 95% CI (−0.00, 0.01), *p* = 0.38, I2 = 3%]. When the patient combined with other diseases, no statistically significant difference in SMI was observed between the HMB group and the control group [MD = −0.37, 95% CI (−1.24, 0.50), *p* = 0.40] (Details can be found in Supplementary materials).

## 4 Discussion

The results of this meta-analysis indicate a positive effect of HMB or HMB-rich nutritional supplements on HGS in individuals with sarcopenia. However, the present study did not demonstrate significant benefits for GS, FM, FFM, and SMI. Sarcopenia is diagnosed based on muscle strength, muscle mass, and physical performance (1, 35). Different measurement methods are employed for muscle strength, muscle mass, and physical performance. For instance, muscle strength can be assessed through HGS (36–38), muscle mass through SMI, FFM, and FM (39–42), and physical performance through GS (43, 44). Presently, the efficacy of HMB for individuals with sarcopenia remains inconclusive. To our knowledge, this study represents the first meta-analysis investigating the effects of supplementing with HMB or HMB-rich nutritional supplements on individuals diagnosed with sarcopenia.

The study by Bear et al. (11) indicates that HMB or supplements containing HMB have beneficial effects on muscle mass and strength in frail individuals. Wu et al.’s (45) research suggests that HMB can improve muscle loss in the elderly. Han et al.’s (46) study found that HMB may help mitigate muscle loss in patients after hip replacement surgery. Results from Martin-Cantero et al.’s (47) research suggest that HMB may effectively improve muscle mass in the elderly. A randomized controlled trial (48) indicates that supplementing with HMB, especially without combining exercise, can enhance muscle mass in the elderly. These studies consistently demonstrate the beneficial effects of HMB on the elderly or frail populations, which aligns with our study results. What sets our study apart is the inclusion of individuals with sarcopenia, analyzing the impact of HMB on muscle strength, muscle mass, and physical performance in this specific population—a distinction not found in other studies. The study by Courel-Ibáñez et al. (13) indicates that HMB does not lead to significant improvements in physical performance, muscle strength, and overall performance in the elderly. A difference from previous meta-analyses is that their intervention involved adding HMB to existing exercise routines, suggesting that physical activity might yield similar or even greater benefits compared to HMB alone (49–51). This is inconsistent with our study results, and the discrepancy may be attributed to differences in the study populations. Regrettably, for sarcopenia patients who face difficulties or are unable to engage in physical activity, sports do not appear to be their optimal choice. Our study results, for the first time, demonstrate that supplementing with HMB or HMB-rich supplements has a certain effect on muscle strength in sarcopenia patients. During the revision process of sarcopenia guidelines, there has been an increasing emphasis on muscle strength. It is recognized that muscle strength is more accurate than muscle mass in predicting adverse outcomes. HGS, as a representative measure of muscle strength, has garnered significant attention in many guidelines and is considered a key marker for evaluating and diagnosing sarcopenia (6, 7). In our study, patients showed improved grip strength after oral intake of HMB- or HMB-rich nutritional supplements. This suggests an enhancement in muscle strength among sarcopenia patients, which can contribute to building confidence and facilitating better rehabilitation. HMB may serve as an effective therapeutic approach for sarcopenia patients, particularly those unable to engage in physical exercise. HMB increases protein synthesis through the mTOR pathway, inhibits catabolic metabolic pathways to reduce protein breakdown, enhances satellite cell proliferation, and decreases the release of inflammatory factors, thereby promoting muscle tissue repair (52–54). Additionally, it increases mitochondrial synthesis and fatty acid oxidation to improve aerobic capacity (55–57). Research indicates (58, 59) a positive correlation between plasma HMB levels and muscle strength as well as muscle mass. Studies (60, 61) also demonstrate that oral HMB supplementation can alleviate the decline in muscle mass in the elderly. HMB may potentially serve as an effective therapeutic drug for treating sarcopenia in clinical settings, offering a promising solution for sarcopenia patients who are unable to engage in physical activity. However, the impact of HMB on sarcopenia patients remains a subject of debate, and further randomized controlled trials are needed to provide high-quality evidence.

The studies included in our analysis exhibit significant heterogeneity, and despite employing various methods to adjust the results, a high degree of heterogeneity persists. Despite our thorough investigation and the application of subgroup analyses and sensitivity analyses, unfortunately, the source of heterogeneity remains unidentified. This may be attributed to the lack of uniform diagnostic criteria for sarcopenia, as well as factors such as ethnicity, gender, nutritional supplements, intervention methods, and dosage. This inference is supported by the fact that the studies included participants from different countries, diagnosed based on specific regional standards. Additionally, there are variations in the gender distribution of study participants, with some studies including only male or female patients, and others including both genders. Furthermore, the nutritional supplement components used in each study vary, and there are differences in intervention methods and the dosage of supplemented HMB. Fortunately, the results of subgroup analyses and sensitivity analyses align with the main outcomes, leading us to believe that the impact of this heterogeneity on the primary outcomes is limited (Details can be found in Supplementary materials).

This meta-analysis has several limitations. Firstly, we included only six studies, as there is a scarcity of randomized controlled trials investigating the use of HMB or HMB-rich nutritional supplements as intervention measures for treating sarcopenia patients. To address this limitation, we incorporated various types of HMB supplements, different control group settings, and diverse sarcopenia patient populations, inevitably increasing the heterogeneity of this study. Additionally, the prevalence and diagnostic criteria for sarcopenia differ between men and women (62), underscoring the need to distinguish the impact of sarcopenia on males and females. However, among the six studies included, five recruited both male and female patients, and unfortunately, there was no differentiation between genders in the results section. Therefore, future research should continue to conduct large-scale, carefully designed randomized controlled trials to provide high-quality evidence.

## 5 Conclusion

Through this study, we have found that HMB or HMB-rich nutritional supplements are beneficial for muscle strength in sarcopenia patients. However, there is limited evidence demonstrating its efficacy on both muscle mass and physical performance in sarcopenia individuals. Furthermore, HMB may be a treatment option for sarcopenia patients, but larger-scale randomized controlled trials are still needed to confirm this conclusion.

## Data availability statement

The original contributions presented in the study are included in the article/Supplementary material, further inquiries can be directed to the corresponding author.

## Author contributions

HS: Formal analysis, Writing – review & editing, Writing – original draft, Data curation. HZ: Data curation, Writing – review & editing. YG: Writing – review & editing, Data curation. SX: Writing – review & editing. WS: Writing – review & editing. XZ: Writing – review & editing. HL: Writing – review & editing. GC: Writing – review & editing. PT: Formal analysis, Investigation, Supervision, Writing – review & editing. JL: Writing – review & editing, Methodology, Supervision, Validation, Funding acquisition.
