# Supplementary material for: The effects of β-hydroxy-β-methylbutyrate or HMB-rich nutritional supplements on sarcopenia patients: a systematic review and meta-analysis
Source: Front Med (Lausanne). 2024 Jul 12;11:1348212. doi: 10.3389/fmed.2024.1348212 (PMC11272589; doi:10.3389/fmed.2024.1348212)
Supplement: Supplementary file 1 [file Data_Sheet_1.docx]

Supplementary Material

# 1 Supplementary Data

Search strategy

Cochrane Library

#1 (Sarcopeni*):ti,ab,kw OR (presarcopeni*):ti,ab,kw OR (pre-sarcopeni*):ti,ab,kw

#2 MeSH descriptor: [Sarcopenia] explode all trees

#3 #1 OR #2

#4 (beta-hydroxy-beta-methylbutyrate):ti,ab,kw

#5 (β-hydroxy-β-methylbutyrate):ti,ab,kw

#6 (HMB):ti,ab,kw

#7 #4 OR #5 OR #6

#8 #3 AND #7

Embase

#1 'sarcopenia'/exp

#2 sarcopeni*:ab,ti OR presarcopeni*:ab,ti OR 'pre sarcopeni*':ab,ti

#3 #1 OR #2

#4 'beta hydroxy beta methylbutyrate':ab,ti OR 'β hydroxy β methylbutyrate':ab,ti OR hmb:ab,ti

#5 #3 AND #4

Web of Science

#1 (((TS=(Sarcopeni*)) OR TS=(Sarcopenia)) OR TS=(presarcopeni*)) OR TS=(pre-sarcopeni*)

#2 ((TS=(β hydroxy β methylbutyrate)) OR TS=(beta hydroxy beta methylbutyrate)) OR TS=(hmb)

#3 #1 AND #2

PubMed

#1 (((Sarcopenia[MeSH Terms]) OR (pre sarcopeni*[Title/Abstract])) OR (presarcopeni*[Title/Abstract])) OR (sarcopeni*[Title/Abstract])

#2 ((beta-hydroxy-beta-methylbutyrate[Title/Abstract]) OR (β-hydroxy-β-methylbutyrate[Title/Abstract])) OR (HMB[Title/Abstract])

#3 #1 AND #2

# 2 Supplementary Figures and Tables

# 2.1 Supplementary Figures


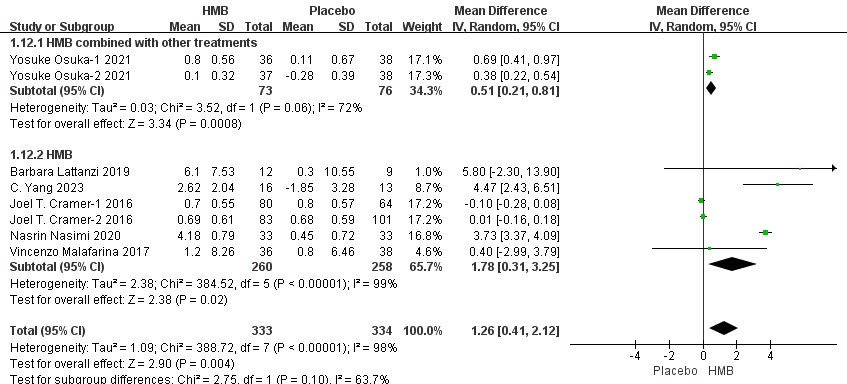


**Supplementary Figure 1：**Forest plot for changes in hand grip strength. The horizontal lines represent 95% CI. The diamond data markers indicate the mean difference (MD) of HMB supplementation on hand grip strength.


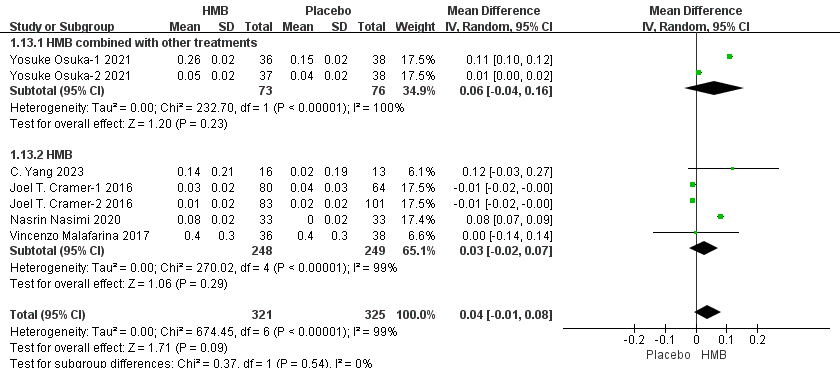


**Supplementary Figure 2：**Forest plot for changes in grip speed. The horizontal lines represent 95% CI. The diamond data markers indicate the mean difference (MD) of HMB supplementation on grip speed.


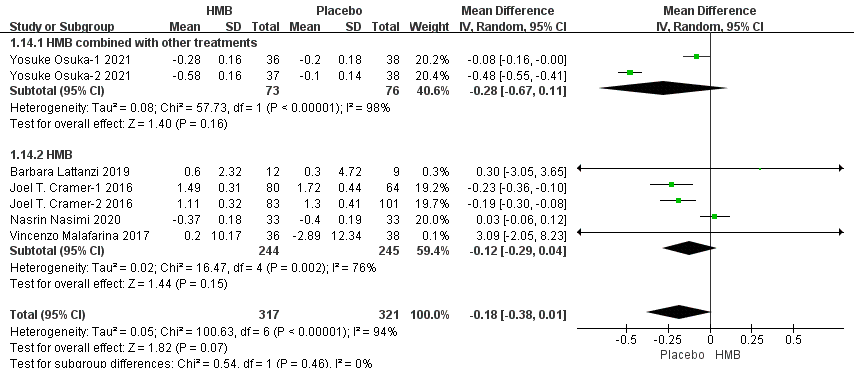


**Supplementary Figure 3：**Forest plot for changes in fat mass. The horizontal lines represent 95% CI. The diamond data markers indicate the mean difference (MD) of HMB supplementation on fat mass.


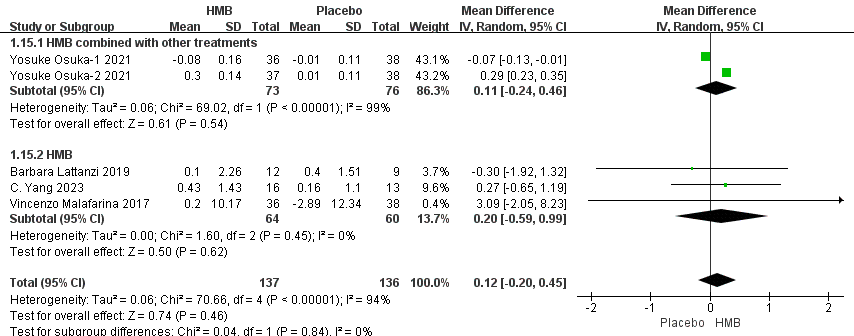


**Supplementary Figure 4：**Forest plot for changes in fat free mass. The horizontal lines represent 95% CI. The diamond data markers indicate the mean difference (MD) of HMB supplementation on fat free mass.


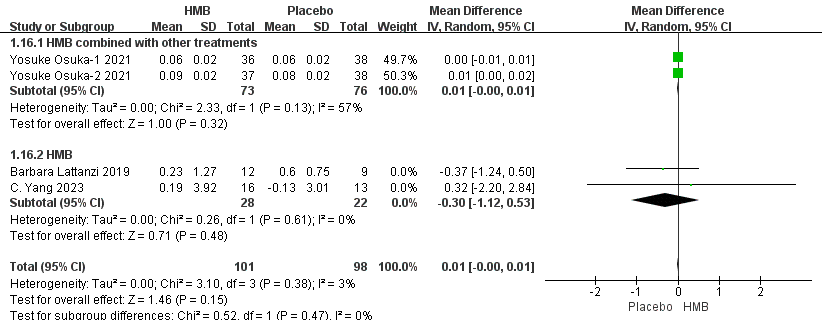


**Supplementary Figure 5：**Forest plot for changes in skeletal muscle index. The horizontal lines represent 95% CI. The diamond data markers indicate the mean difference (MD) of HMB supplementation on skeletal muscle index.


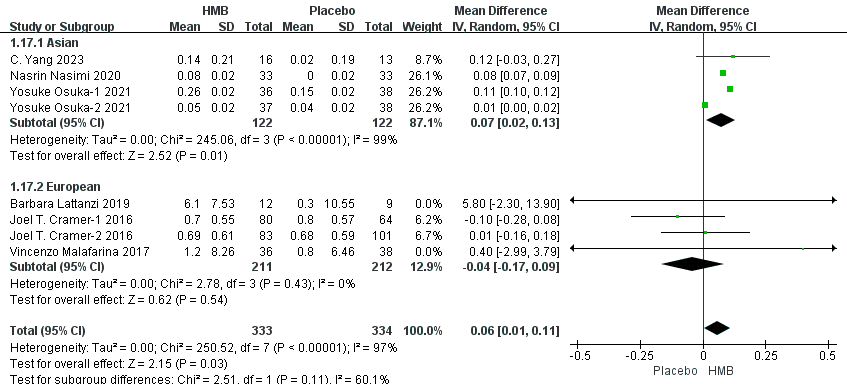


**Supplementary Figure 6：**Forest plot for changes in hand grip strength. The horizontal lines represent 95% CI. The diamond data markers indicate the mean difference (MD) of HMB supplementation on hand grip strength.


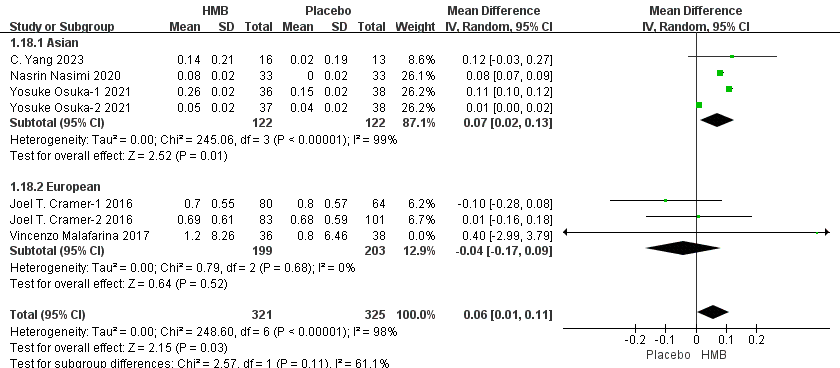


**Supplementary Figure 7：**Forest plot for changes in grip speed. The horizontal lines represent 95% CI. The diamond data markers indicate the mean difference (MD) of HMB supplementation on grip speed.


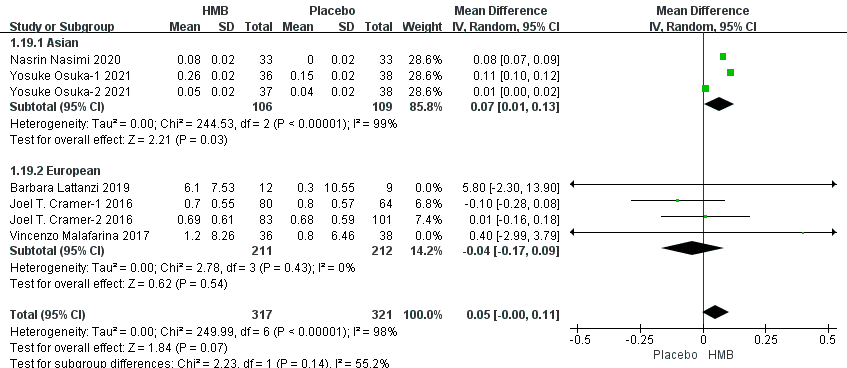


**Supplementary Figure 8：**Forest plot for changes in fat mass. The horizontal lines represent 95% CI. The diamond data markers indicate the mean difference (MD) of HMB supplementation on fat mass.


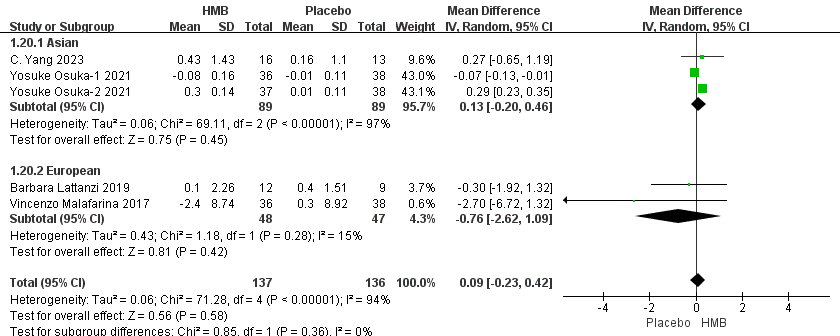


**Supplementary Figure 9：**Forest plot for changes in fat free mass. The horizontal lines represent 95% CI. The diamond data markers indicate the mean difference (MD) of HMB supplementation on fat free mass.


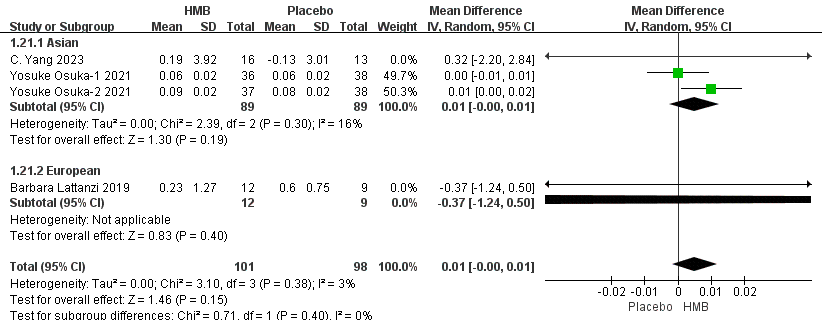


**Supplementary Figure 10：**Forest plot for changes in skeletal muscle index. The horizontal lines represent 95% CI. The diamond data markers indicate the mean difference (MD) of HMB supplementation on skeletal muscle index.


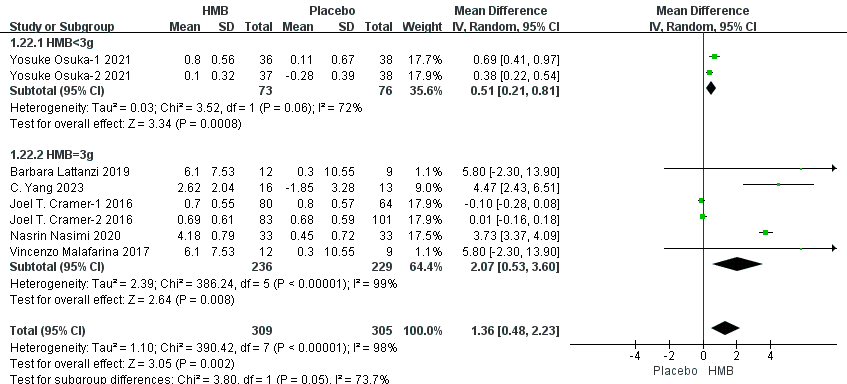


**Supplementary Figure 11：**Forest plot for changes in hand grip strength. The horizontal lines represent 95% CI. The diamond data markers indicate the mean difference (MD) of HMB supplementation on hand grip strength.


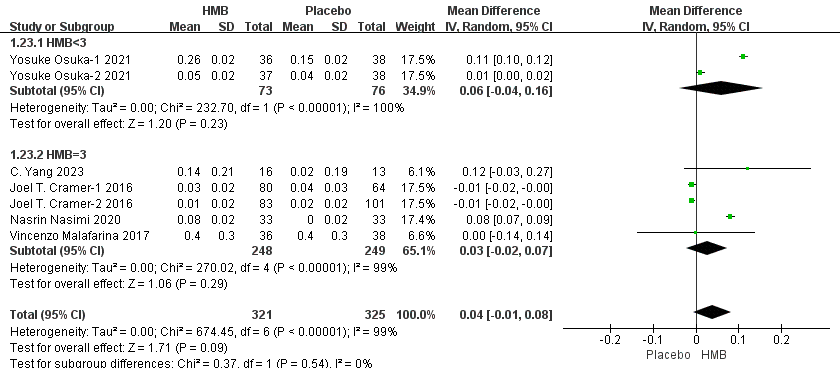


**Supplementary Figure 12：**Forest plot for changes in grip speed. The horizontal lines represent 95% CI. The diamond data markers indicate the mean difference (MD) of HMB supplementation on grip speed.


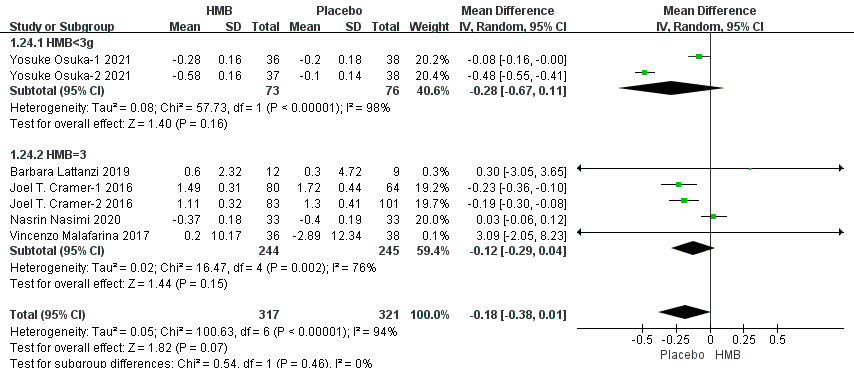


**Supplementary Figure 13：**Forest plot for changes in fat mass. The horizontal lines represent 95% CI. The diamond data markers indicate the mean difference (MD) of HMB supplementation on fat mass.


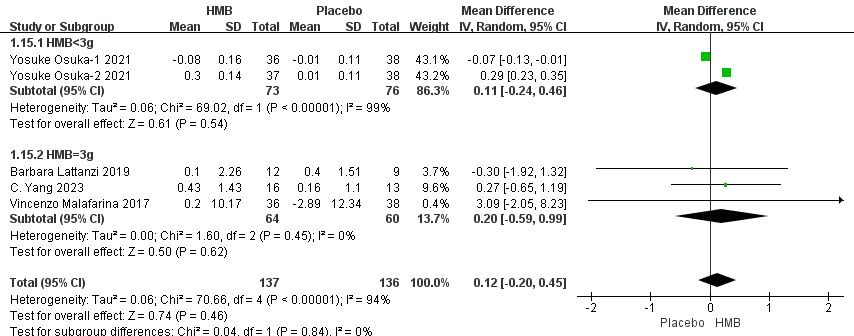


**Supplementary Figure 14：**Forest plot for changes in fat free mass. The horizontal lines represent 95% CI. The diamond data markers indicate the mean difference (MD) of HMB supplementation on fat free mass.


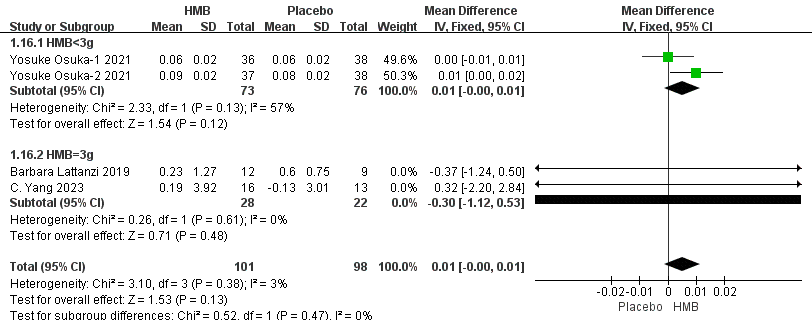


**Supplementary Figure 15：**Forest plot for changes in skeletal muscle index. The horizontal lines represent 95% CI. The diamond data markers indicate the mean difference (MD) of HMB supplementation on skeletal muscle index.


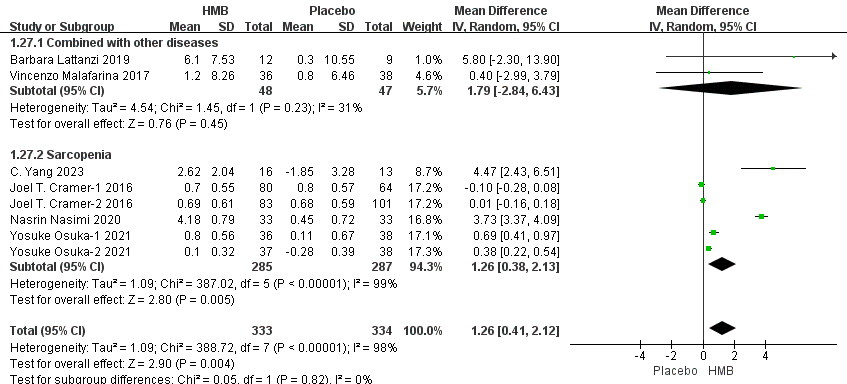


**Supplementary Figure 16：**Forest plot for changes in hand grip strength. The horizontal lines represent 95% CI. The diamond data markers indicate the mean difference (MD) of HMB supplementation on hand grip strength.


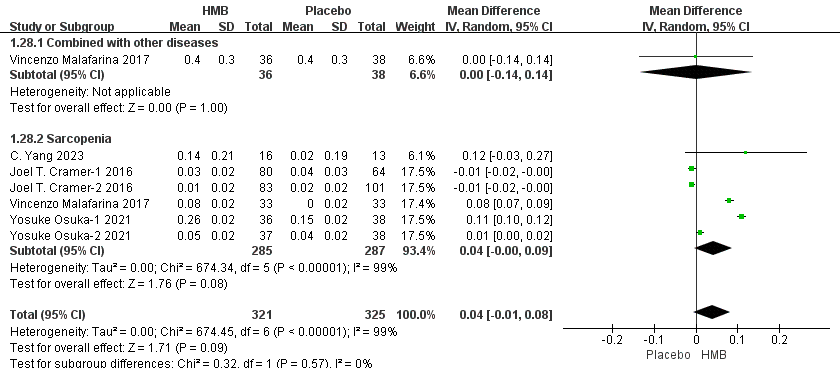


**Supplementary Figure 17：**Forest plot for changes in grip speed. The horizontal lines represent 95% CI. The diamond data markers indicate the mean difference (MD) of HMB supplementation on grip speed.


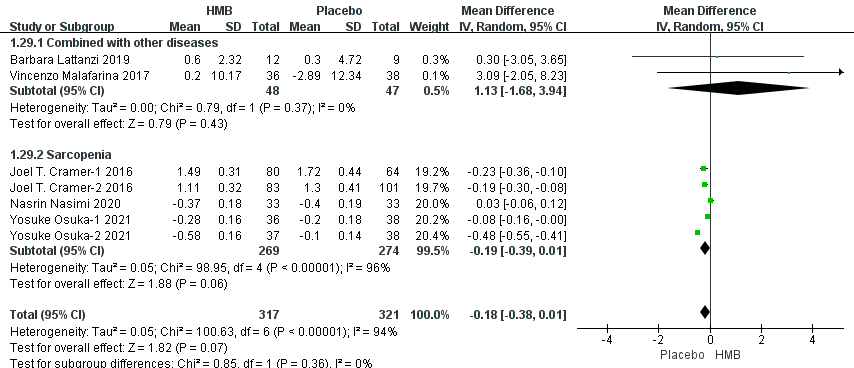


**Supplementary Figure 18：**Forest plot for changes in fat mass. The horizontal lines represent 95% CI. The diamond data markers indicate the mean difference (MD) of HMB supplementation on fat mass.


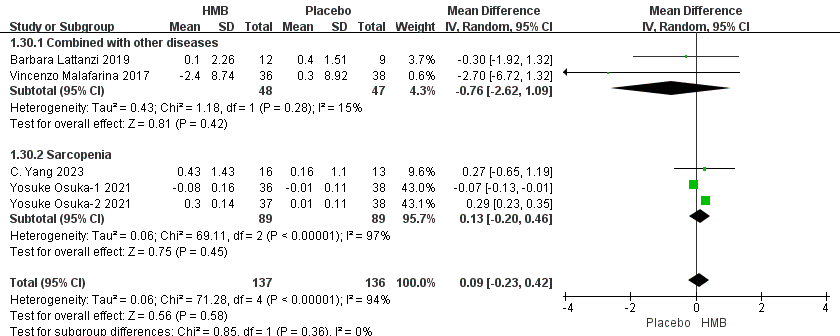


**Supplementary Figure 19：**Forest plot for changes in fat free mass. The horizontal lines represent 95% CI. The diamond data markers indicate the mean difference (MD) of HMB supplementation on fat free mass.


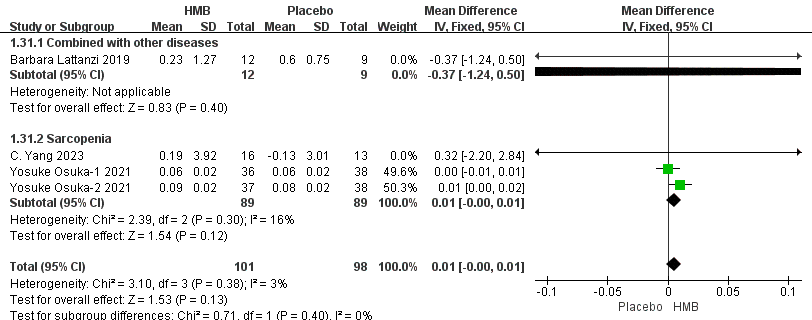


**Supplementary Figure 20：**Forest plot for changes in skeletal muscle index. The horizontal lines represent 95% CI. The diamond data markers indicate the mean difference (MD) of HMB supplementation on skeletal muscle index.

# 2.2 Supplementary Tables

| Sensitivity analysis for FM | | | |
| --- | --- | --- | --- |
| Study | MD(95%CI) | P | I^2^ |
| Barbara Lattanzi 2019 | -0.19 [-0.38, 0.01] | P=0.07 | I^2^=95% |
| Joel T. Cramer-1 2016 | -0.17 [-0.41, 0.07] | P=0.16 | I^2^=95% |
| Joel T. Cramer-2 2016 | -0.18 [-0.43, 0.07] | P=0.15 | I^2^=95% |
| Nasrin Nasimi 2020 | -0.24 [-0.44, -0.03] | P=0.02 | I^2^=92% |
| Yosuke Osuka-1 2021 | -0.21 [-0.45, 0.04] | P=0.1 | I^2^=94% |
| Yosuke Osuka-2 2021 | -0.11 [-0.22, -0.00] | P=0.05 | I^2^=70% |
| Vincenzo Malafarina 2017 | -0.19 [-0.39, 0.01] | P=0.06 | I^2^=95% |

| Sensitivity analysis for GS | | | |
| --- | --- | --- | --- |
| Study | MD(95%CI) | P | I^2^ |
| C. Yang 2023 | 0.03 [-0.01, 0.08] | P=0.15 | I^2^=99% |
| Joel T. Cramer-1 2016 | 0.05 [-0.00, 0.10] | P=0.07 | I^2^=99% |
| Joel T. Cramer-2 2016 | 0.05 [-0.00, 0.10] | P=0.06 | I^2^=99% |
| Nasrin Nasimi 2020 | 0.03 [-0.02, 0.08] | P=0.23 | I^2^=99% |
| Yosuke Osuka-1 2021 | 0.02 [-0.01, 0.06] | P=0.24 | I^2^=98% |
| Yosuke Osuka-2 2021 | 0.05 [-0.01, 0.10] | P=0.1 | I^2^=99% |
| Vincenzo Malafarina 2017 | 0.04 [-0.00, 0.09] | P=0.08 | I^2^=99% |

| Sensitivity analysis for HGS | | | |
| --- | --- | --- | --- |
| Study | MD(95%CI) | P | I^2^ |
| Barbara Lattanzi 2019 | 1.22[0.36, 2.07] | P=0.005 | I^2^=98% |
| C. Yang 2023 | 0.96 [0.08, 1.83] | P=0.03 | I^2^=98% |
| Joel T. Cramer-1 2016 | 1.59 [0.53, 2.66] | P=0.003 | I^2^=98% |
| Joel T. Cramer-2 2016 | 1.58 [0.49, 2.68] | P=0.005 | I^2^=98% |
| Nasrin Nasimi 2020 | 0.37 [0.00, 0.74] | P=0.05 | I^2^=88% |
| Yosuke Osuka-1 2021 | 1.41 [0.40, 2.42] | P=0.006 | I^2^=98% |
| Yosuke Osuka-2 2021 | 1.53 [0.37, 2.68] | P=0.01 | I^2^=98% |
| Vincenzo Malafarina 2017 | 1.31[0.43,2.18] | P=0.003 | I^2^=98% |

| Sensitivity analysis for FFM | | | |
| --- | --- | --- | --- |
| Study | MD(95%CI) | P | I^2^ |
| Barbara Lattanzi 2019 | 0.11 [-0.22, 0.44] | P=0.53 | I^2^=96% |
| C. Yang 2023 | 0.07 [-0.27, 0.42] | P=0.68 | I^2^=96% |
| Yosuke Osuka-1 2021 | 0.29 [0.23, 0.35] | P＜0.00001 | I^2^=0% |
| Yosuke Osuka-2 2021 | -0.07 [-0.13, -0.01] | P=0.03 | I^2^=0% |
| Vincenzo Malafarina 2017 | 0.11 [-0.21, 0.43] | P=0.5 | I^2^=96% |

| Sensitivity analysis for SMI | | | |
| --- | --- | --- | --- |
| Study | MD(95%CI) | P | I^2^ |
| Barbara Lattanzi 2019 | 0.01 [-0.00, 0.01] | P=0.12 | I^2^=16% |
| C. Yang 2023 | 0.01 [-0.00, 0.01] | P=0.13 | I^2^=34% |
| Yosuke Osuka-1 2021 | 0.01 [0.00, 0.02] | P=0.03 | I^2^=0% |
| Yosuke Osuka-2 2021 | -0.00 [-0.01, 0.01] | P=0.99 | I^2^=0% |
